# Molecular characterization of a *Trichinella spiralis* enolase and its interaction with the host’s plasminogen

**DOI:** 10.1186/s13567-019-0727-y

**Published:** 2019-12-05

**Authors:** Peng Jiang, You Jiao Zao, Shu Wei Yan, Yan Yan Song, Dong Min Yang, Li Yuan Dai, Ruo Dan Liu, Xi Zhang, Zhong Quan Wang, Jing Cui

**Affiliations:** Department of Parasitology, Medical College, Zhengzhou University, Zhengzhou, 450052 China

## Abstract

The binding and activation of host plasminogen (PLG) by worm surface enolases has been verified to participate in parasite invasion, but the role of this processes during *Trichinella spiralis* infection has not been clarified. Therefore, the expression and immunolocalization of a *T. spiralis* enolase (TsENO) and its binding activity with PLG were evaluated in this study. Based on the three-dimensional (3D) molecular model of TsENO, the protein interaction between TsENO and human PLG was analysed by the ZDOCK server. The interacting residues were identified after analysis of the protein–protein interface by bioinformatics techniques. The key interacting residues were confirmed by a series of experiments. The qPCR analysis results demonstrated that Ts-*eno* was transcribed throughout the whole life cycle of *T. spiralis*. The immunofluorescence assay (IFA) results confirmed that TsENO was distributed on the *T. spiralis* surface. The binding assays showed that recombinant TsENO (rTsENO) and native TsENO were able to bind PLG. Four lysine residues (90, 289, 291 and 300) of TsENO were considered to be active residues for PLG interaction. The quadruple mutant (Lys90Ala + Lys289Ala + Lys291Ala + Lys300Ala) TsENO, in which the key lysine residues were substituted with alanine (Ala) residues, exhibited a reduction in PLG binding of nearly 50% (45.37%). These results revealed that TsENO has strong binding activity with human PLG. The four lysine residues (90, 289, 291 and 300) of TsENO play an important role in PLG binding and could accelerate PLG activation and invasion of the host’s intestinal wall by *T. spiralis*.

## Introduction

Plasminogen (PLG) circulates throughout the endovascular network and serves as a zymogen that initiates the process of fibrinolysis [1, 2]. PLG is synthesized by several organs/tissues, mainly the liver and simultaneously by the kidney, brain, heart, lung, spleen, and gut; in addition, a considerable quantity is found in extravascular fluids [3, 4]. The critical and final step in the process of fibrinolytic system activation is PLG activation; this activation is usually dependent on activators that convert PLG into its active form, plasmin (PLM) [5–8]. PLG and PLM play fundamental roles in fibrinolysis and the degradation of structures related to tissue repair and immunity, such as fibrin clots and the extracellular matrix (ECM) [8, 9]. Evidently, the activation of PLG is utilized by some pathogens to penetrate the tissue barrier and facilitate invasion [10].

The interaction between PLG and pathogens is usually regulated by various and ubiquitous PLG receptors [11–13]. A fairly large number of pathogens, including bacteria, fungi and parasites, exhibit surface expression of PLG receptors that immobilize PLG, resulting in its activation; it has been proposed that the activation of PLG facilitates the migration and invasion of these pathogens to different tissues in the host [14–17]. To date, more than 85 different kinds of PLG receptors have been identified to play roles in infectious diseases [18]. Among these receptors, enolase is perhaps the most studied PLG-binding protein in different organisms and is frequently exploited by parasites [18–21]. Enolase is not only a glycolytic enzyme but also a multifunctional protein [20]. As a surface-expressed PLG receptor, enolase is able to mediate the activation of PLG and the degradation of the ECM [21].

Trichinellosis is an important food-borne zoonosis caused by the ingestion of raw or undercooked meat contaminated with viable, infective *T. spiralis* larvae. The critical variable in the establishment of *T. spiralis* infection is whether the larvae invade the intestinal mucosa to develop further. However, the mechanism of larval invasion is unclear to date [22–24]. Our previous LC–MS/MS results of infective larval proteins showed that *T. spiralis* enolase (TsENO) was present at very high levels after co-culture with intestinal epithelial cells (IECs) in vitro [25]. The relative transcription level of the *T. spiralis* enolase gene (Ts-*eno*) was upregulated in larvae cultured with IECs [26]. High expression of TsENO was also identified in a screen of *T. spiralis* intestinal infective larvae (IIL) surface proteins [27].

Previous studies indicate that TsENO may bind the host’s PLG to activate the fibrinolytic system, degrade the ECM, and promote larval penetration of the tissue barrier during invasion. For the purpose of further understanding the relationship between *T. spiralis* and its host, bioinformatics methods and experimental techniques were used to explore the interaction between TsENO and the host’s PLG in this work.

## Materials and methods

### Bioinformatics analysis, molecular modelling and model evaluation of TsENO

The whole coding cDNA sequence (CDS) of TsENO (Tsp_09466) was retrieved from GenBank (Accession no. XM_003371185). The three-dimensional (3D) molecular structure of full-length human PLG (X-ray crystal structure; ID: 4DUR) was obtained from the Protein Data Bank (PDB). The background biochemical characteristics of TsENO were analysed by bioinformatics software and web servers. Physical and chemical parameters of the TsENO protein were predicted with the ProtParam tool [28]. SignalP Server (version 4.1) was used to predict the cleavage sites in the signal peptide [29]. A molecular model of TsENO was generated by the threading protein server Iterative Threading ASSEmbly Refinement (I-TASSER) [30]. The 3D structure and the quality of the models were evaluated and verified by SAVES v5.0 [31–35].

### Protein–protein docking

The ZDOCK (version 3.0.2) algorithm with the default parameters was used to predict the structures of the TsENO-PLG complex. ZDOCK was used to search and analyse all possible binding poses between TsENO and PLG in 3D space and to assess every binding pose by an energy-based scoring function [36]. The docking results were visualized by visual molecular dynamics (VMD) [37].

### Analyses of protein–protein docking

TsENO-PLG interaction plots were generated by the DIMPLOT algorithm in LigPlot+ (version 2.1) [38]. To check the involvement of TsENO-interacting residues obtained from DIMPLOT, the H-bonding and hydrophobic interactions between TsENO and PLG and the loss of accessible surface area (ASA) and solvation energy (Δ^i^G) of the interfacing residues were re-checked and confirmed by PDBe PISA (version 1.52) [39]. 3D structural alignments between TsENO and its variants were generated by SuperPose (version 1.0) and Swiss-PdbViewer (DeepView, version 4.1) [40, 41].

### Expression and purification of TsENO and its site-directed mutant

The coding sequences for TsENO and its site-specific mutant sequence were chemically synthesized by Sangon Biotech Co., Ltd. (Shanghai, China) and introduced into *Escherichia coli*. The synthesized coding sequences harbouring BamHI and PstI restriction enzyme sites were cloned into the prokaryotic expression vector pQE-80L with one step cloning kit (Vazyme, Nanjing, China). Recombinant pQE-80L/TsENO and its variant were transformed into *E. coli* BL21 (DE3) (Novagen, La Jolla, USA). The expression of rTsENO and its mutant (M-rTsENO) was induced for 5 h at 30 °C by using 0.5 mM isopropyl β-d-1-thiogalactopyranoside (IPTG). The rTsENO and M-rTsENO proteins were purified using a Ni–NTA sefinose™ resin kit (Sangon Biotech, Shanghai, China). The concentrations of the purified proteins were measured by the bicinchoninic acid (BCA) method, and the recombinant proteins were then analysed by SDS-PAGE with 12% acrylamide separating gels.

### Preparation of anti-rTsENO and M-rTsENO antibodies

Fifteen BALB/c mice (female, 6 weeks old, purchased from the Experimental Animal Center of Henan Province) were vaccinated with rTsENO. The rTsENO protein was emulsified in Freund’s complete adjuvant and was subcutaneously injected into the abdomen of the BALB/c mice (20 μg rTsENO per mouse). All mice were boosted twice by using the same amount of rTsENO (emulsified in Freund’s incomplete adjuvant) at intervals of 10 days. Blood samples (50 µL/mouse) from vaccinated mice were collected 10 days after the final immunization. The anti-rTsENO antibody titre in serum was measured by ELISA. The serum anti-mutated rTsENO antibody titre was measured by the same protocol.

### Real-time quantitative PCR (qPCR) analysis

The *T. spiralis* isolate (ISS534) used in this study was maintained in BALB/c mice by oral inoculation in our laboratory. Total RNA of *T. spiralis* muscle larvae (ML), IIL, 3-day-old adult worms (3 days AW), 6 days AW and newborn larvae (NBL) were extracted by Trizol (Invitrogen™, Carlsbad, USA), and the RNA quality was assessed by agarose gel electrophoresis and ultramicrospectrophotometry (NanoDrop 2000, Thermo Scientific, Wilmington, USA). Then, RNA was transcribed to cDNA by using an RNA PrimeScript™ RT reagent kit containing DNase I (with gDNA Eraser, Takara, Japan), which can digest single- and double-stranded DNA [42]. Primer Premier 5 was used to design the primers for Ts-*eno* (5′-AAACGGCGGTTCTCACGCAG-3′; 5′-TCGGCGCAAATCCACCTTCG-3′). The housekeeping gene glyceraldehyde-3-phosphate dehydrogenase (GAPDH) was used as the standard control (5′-AGATGCTCCTATGTTGGTTATGGG-3′; 5′-GTCTTTTGGGTTGCCGTTGTAG-3′). Fifty nanograms of cDNA in a 20 µL reaction volume was used to quantify the transcriptional level of Ts-*eno* by using a 7500 Fast Real-Time PCR System (Applied Biosystems, Foster City, USA) as previously described [23, 26]. All experiments were repeated three times. The two amplicons of Ts-*eno* and GAPDH were examined by 2% agarose gel electrophoresis. The melt curves of Ts-*eno* and GAPDH were generated by 7500 Software (version 2.0.5 for 7500 Fast Real-Time PCR Products, Applied Biosystems, USA). The comparative Ct method (2^−∆∆Ct^) was used to determine the normalized transcription level of Ts-*eno* [25, 43].

### Immunolocalization of TsENO

An indirect immunofluorescence assay (IFA) was used to identify whether TsENO was expressed on the surface of *T. spiralis* worms at different stages (ML, 6 h IIL, 24 h IIL, 3 days AW, 6 days AW and NBL) as previously described [44, 45]. Whole worms were blocked in 5% goat serum diluted with PBS for 1 h and were then incubated with a 1:10 dilution of anti-rTsENO serum in a moist chamber at 37 °C for 1 h. At the same time, serum from *T. spiralis*-infected mice and normal mouse serum were utilized as the positive control and negative control, respectively. After three rinses with PBS, worms were incubated with FITC-labelled goat anti-mouse IgG (Santa Cruz, USA) diluted 1:100 for 1 h at 37 °C. Following five washes in PBS, the parasites were finally examined under a fluorescence microscope (Olympus, Japan).

### Analysis of PLG binding by Western blotting

For Western blot analysis, purified rTsENO, purified M-rTsENO, ML soluble antigens, ML excretory-secretory (ES) antigens, *S. cerevisiae* enolase (ScENO; positive control; Sigma-Aldrich, Saint Louis, USA) and BSA (negative control) were loaded into the lanes of a 12% SDS-PAGE gel and electrophoresed. Then, the proteins were transferred to an NC membrane (Millipore, USA), which was blocked with 5% skim milk in TBST at room temperature for 2 h. After washing three times with TBST, the membrane was then incubated with 25 μg/mL human PLG (Sigma-Aldrich) in PBST at 37 °C for 3 h, washed three times with TBST, and incubated with a sheep anti-human PLG antibody (1:2500; Invitrogen™) in TBST for 2 h at room temperature. After washing with TBST, the membrane was incubated with rabbit anti-sheep IgG-HRP (1:10 000; Invitrogen™) for 2 h at room temperature. After washing three times with TBST, the strips were developed using the 3-amino-9-ethylcarbazole (AEC; Solarbio, Beijing, China) substrate.

### Analysis of PLG binding by indirect ELISA

To compare the PLG binding ability between rTsENO and M-rTsENO, an ELISA plate binding assay was adopted. In brief, the ELISA plate was coated with 0.6 μg/well purified rTsENO and incubated overnight at 4 °C. After being washed with PBST three times, the plate was blocked with 5% skim milk for 2 h at 37 °C. The plate was washed three times again, and increasing concentrations of human PLG (0.05, 0.1, 0.2, 0.4, 0.8, 1.2, 1.6, and 2.0 μg/mL in PBST) were then added in duplicate and incubated for 3 h at 37 °C. After washing, the plates were incubated with a sheep anti-human PLG antibody (1:2500; 100 μL/well) at 37 °C for 2 h. After washing, rabbit anti-sheep IgG-HRP was added to the wells (1:10 000; 100 μL/well), and the plates were incubated at 37 °C for 1 h. Finally, the compound o-phenylenediamine dihydrochloride (OPD; Sigma-Aldrich) was used as the substrate for HRP. The optical density (OD) at 450 nm was measured in an ELISA reader (Tecan Schweiz AG, Mannedorf, Switzerland). The wells coated with M-rTsENO (0.6 μg/well), ScENO (0.4 μg/well, positive control) and BSA (0.8 μg/well, negative control) were analysed by the same procedures. All experiments in this part were repeated three times.

### Statistical analysis

The data were analysed by IBM SPSS Statistics 21.0 for Windows (IBM Corporation, NY, USA). The differences in the relative Ts-*eno* expression levels across the different stages were analysed with one-way ANOVA. The intra- and inter-group differences in OD values were assessed by Student’s *t* test and one-way ANOVA as appropriate. The variation trend in the OD values with increasing PLG concentrations was evaluated by a linear algorithm in an ANOVA model. A significance level of *P* < 0.05 was regarded as statistically significant.

## Results

### Biochemical characteristics and molecular model of TsENO

**Figure 1:**
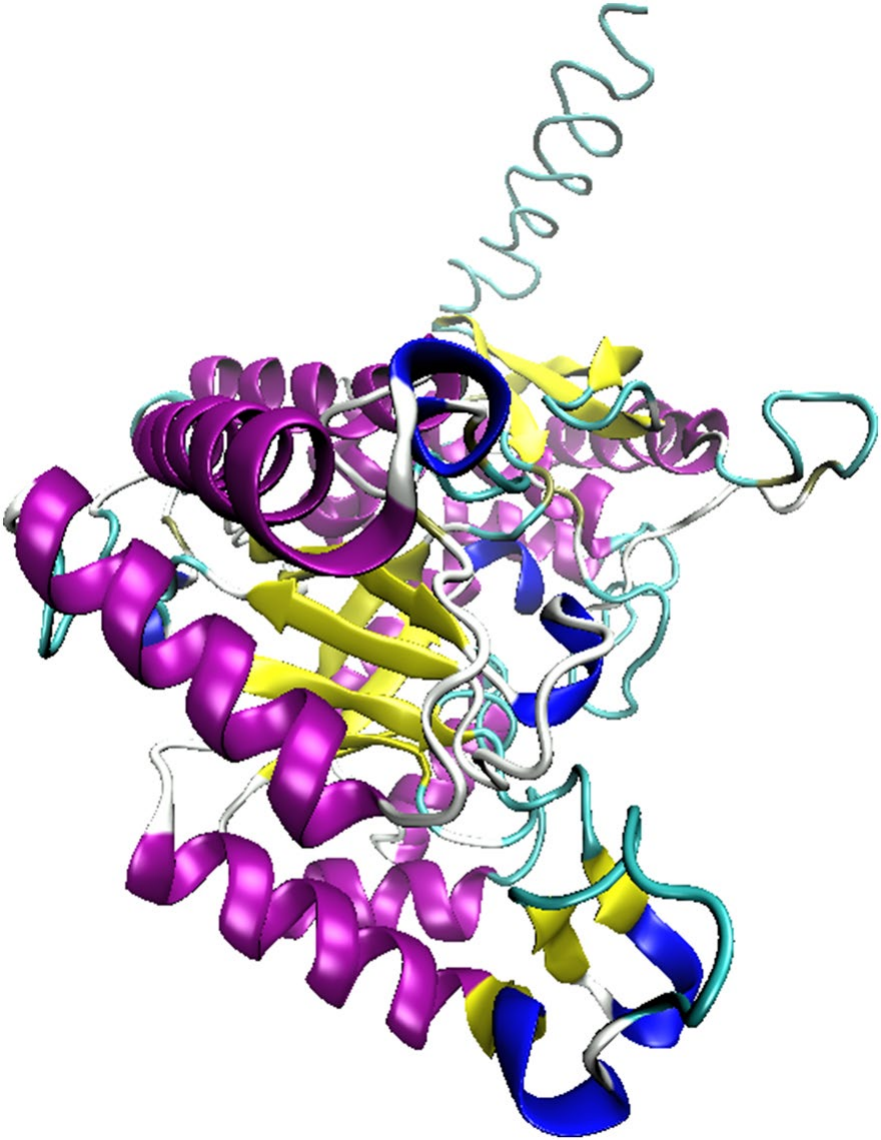
**Cartoon representation of TsENO molecular model.** Secondary structures are coloured differently, with alpha helices in purple, 3_10_helices in blue, extended beta strands in yellow, bridge beta strands in tan, coils in white, and turns in cyan.

**Figure 2:**
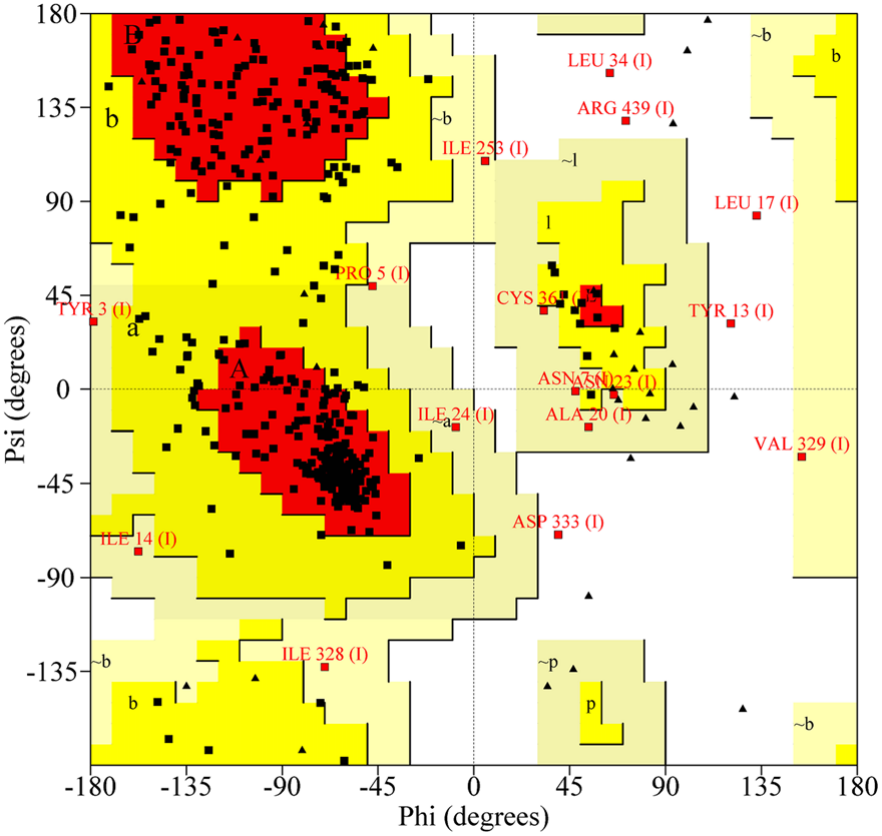
**Ramachandran plot analysis of the TsENO molecular model.** All residues except Gly and Pro are shown as square dots located in the most favoured regions (78.1% in the red area), additional allowed regions (18.3% in the yellow area), generously allowed regions (2.4% in the yellow-grey area) and disallowed regions (1.2% in the white area).

The complete CDS of *T. spiralis* enolase (XM_003371185) contains 1461 bp and encodes 486 amino acids (aa). The SignalP 4.1 prediction showed that there is a signal peptide with a cleavage site between the aa at pos. 13 and 14. The length of the mature protein of TsENO is 473 aa; the molecular mass is 51.95 kDa, and the theoretical isoelectric point (pI) is 5.88. For the aa sequence of TsENO, the I-TASSER server generated and reported up to five models that correspond to the states with the largest partition function (or lowest free energy). Among the top 5 models, the best model, with the highest C-score, was selected as the final molecular model of TsENO (Figure 1). The selected TsENO model was further evaluated by the SAVES v5.0 program. According to the Verify 3D results, 82.24% of the residues had a score of ≥ 0.2 in the 3D-1D profile. The overall quality factor was 90.753 by the ERRAT results. The model quality also passed the PROVE, WHATCHECK and PROCHECK evaluations. The Ramachandran plot revealed that 78.1% of the TsENO residues were in the most favoured region, 20.7% were in allowed regions, and only 1.2% were in disallowed regions, suggesting the validity of the TsENO model (Figure 2).

### Molecular docking between TsENO and PLG

**Figure 3:**
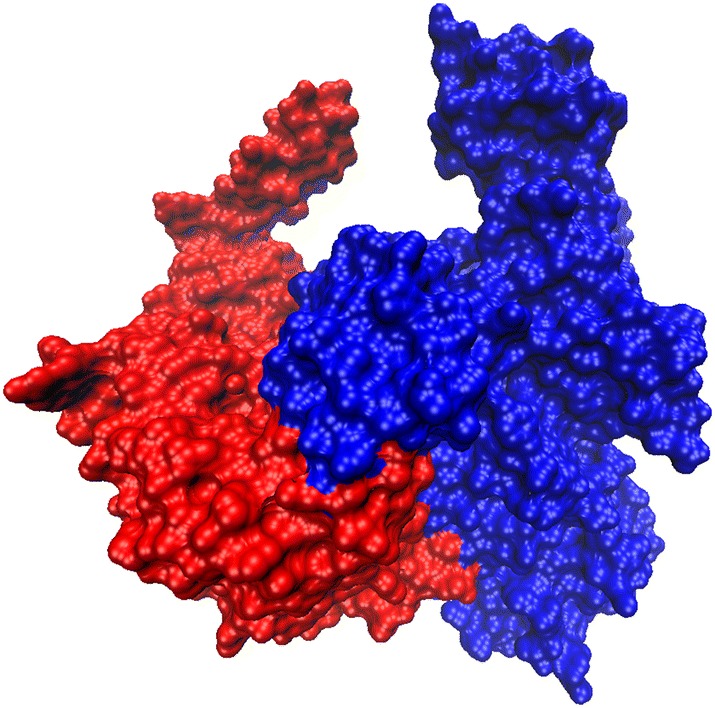
**Protein-protein docking of TsENO and human PLG.** Binding proteins are shown as surface representations. TsENO is shown in red, whereas human PLG (4DUR) is shown in blue.

Via the ZDOCK program, every possible TsENO-PLG binding pose in 3D space calculated and evaluated, and each pose was scored using an energy-based scoring function. The results of the top 2000 binding poses were filtered using a 6 angstrom (Å) distance cutoff for the interacting residues. The reserved predictions were sorted by the ZDOCK score, and the top prediction was selected and further analysed. The ZDOCK protein–protein docking results indicated that TsENO and PLG exhibited surface complementarity in the interface area (Figure 3).

### PLG-interacting residues of TsENO

**Figure 4:**
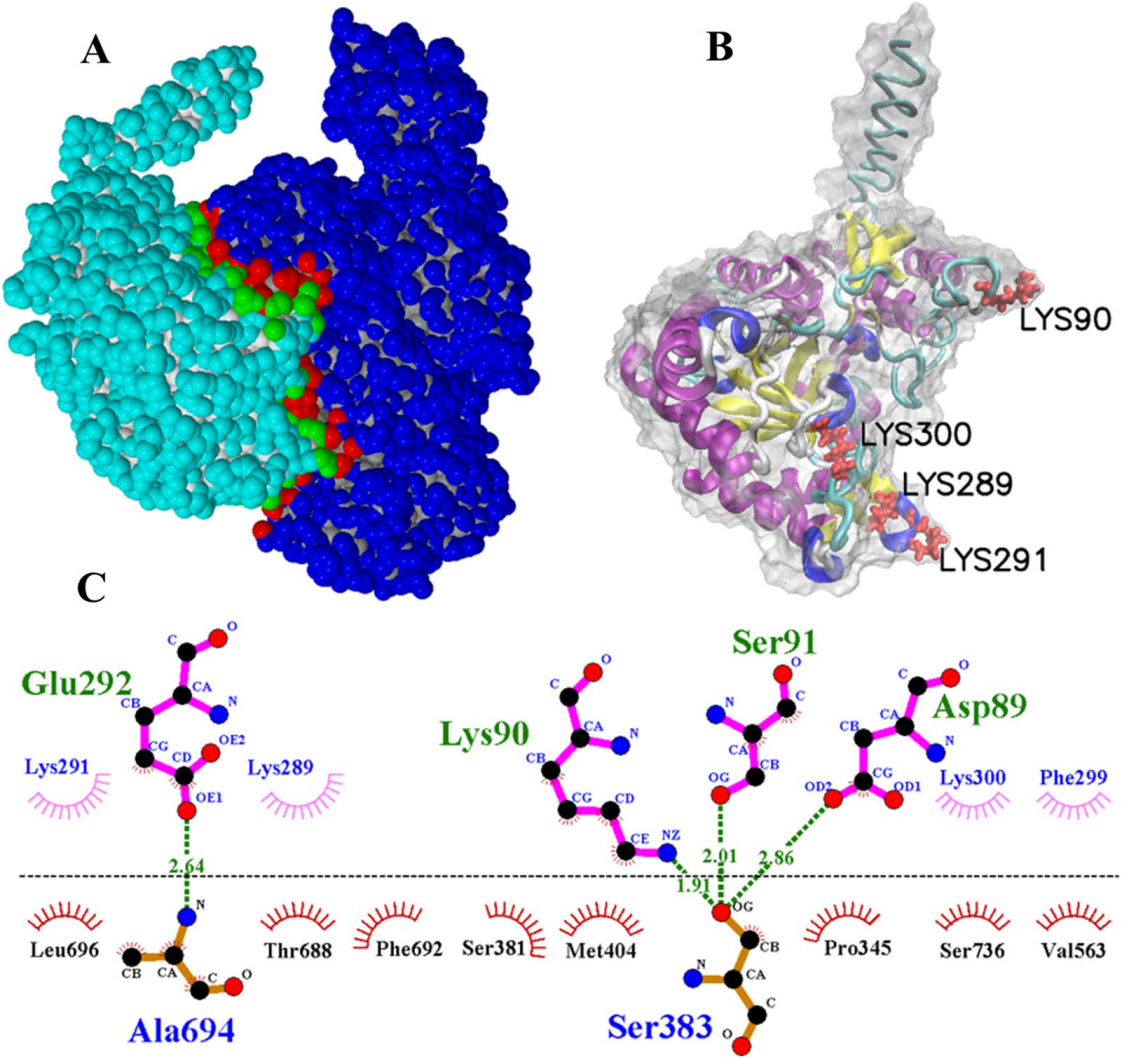
**The interaction between TsENO and human PLG. A** TsENO and PLG bound to each other at the interface region. TsENO is shown in cyan, and PLG is shown in blue. Interface-located residues of TsENO are shown in green, and those of PLG are shown in red. **B** The important residues (Lys90, Lys289, Lys291 and Lys300) for PLG binding are shown in red in the Corey-Pauling-Koltun (CPK) model. **C** TsENO-PLG interaction plot. TsENO residues are shown above the dashed line, and PLG residues are shown in different colours beneath the dashed line. Hydrogen bonds are shown as green dashed lines and labelled with the bond length (Å). The arcs represent the other residues involved in the TsENO-PLG interaction.

**Figure 5:**
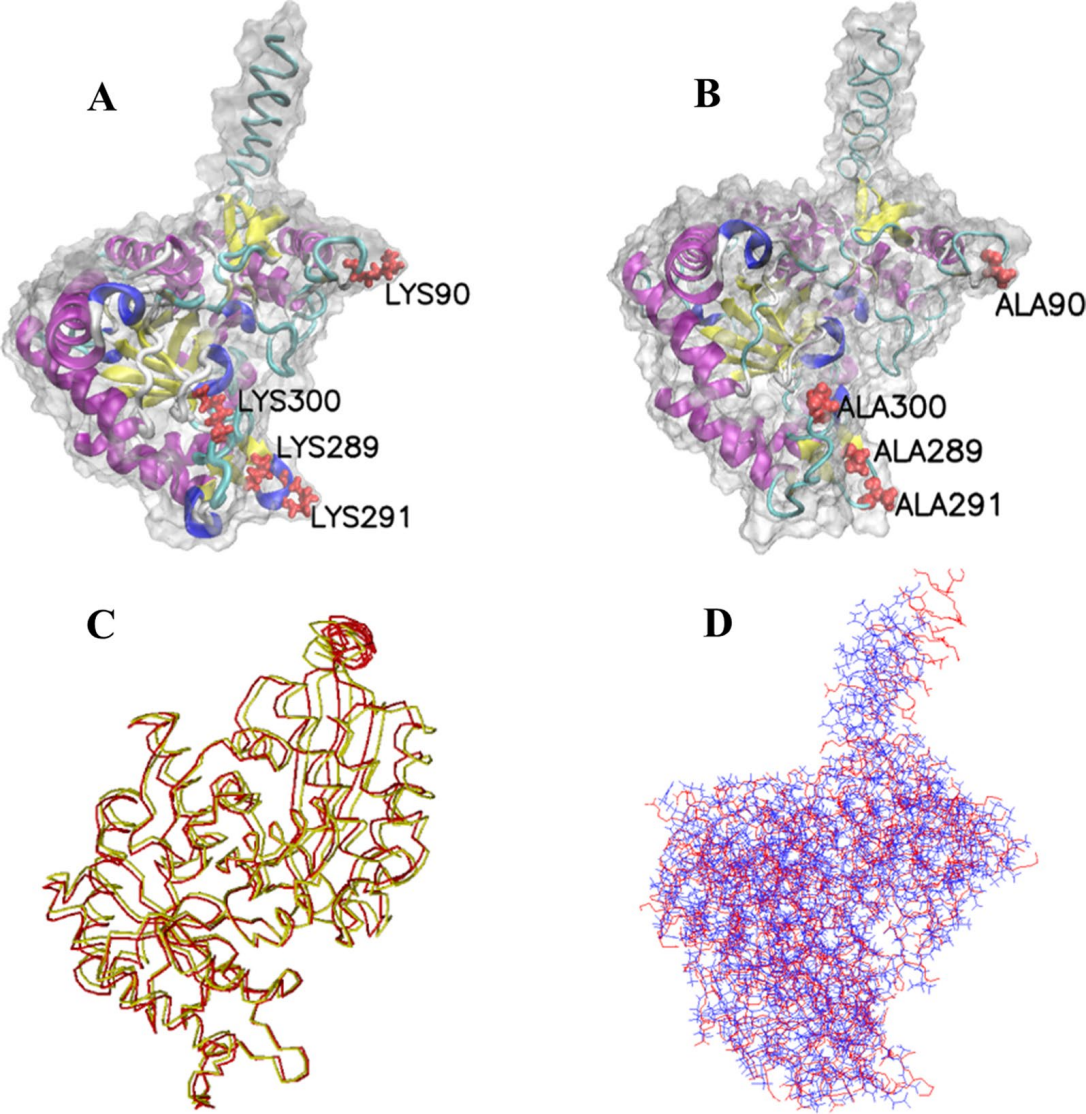
**Structural alignments between TsENO and M-TsENO. A** The interacting residues (Lys90, Lys289, Lys291 and Lys300) of wild-type TsENO are shown in red in the CPK model. **B** Molecular model of M-TsENO constructed by I-TASSER. The site-directed mutated residues (Ala90, Ala289, Ala291 and Ala300) of M-TsENO are shown in red in the CPK model. **C** Superposition of the TsENO (green) and M-TsENO (red) backbones. A local RMSD of 2.63 Å was calculated when aligned over 473 residues. **D** Global 3D structural alignments between TsENO (blue) and M-TsENO (red) showed a global RMSD of 3.30 Å.

The hydrophobic effect and H-bond interaction between TsENO and PLG were analysed by PDBe PISA v1.52 and DIMPLOT. The changes in the accessible surface area (ASA) and the solvation energy effect (Δ^i^G) were calculated by PDBe PISA. Seven lysine residues (90, 198, 229, 233, 289, 291 and 300) were identified in the interface area, and lysine was deemed to be the most frequently appearing interacting amino acid (Table 1, Figure 4A). Lys90 and Lys198 formed H-bonds with PLG, but the ASA loss for Lys198 was only 6.95 Å^2^. The interface showed that these lysine residues, except Lys198, had hydrophobic interactions with PLG. According to the simulated calculation, after 4 of the interacting lysine residues (90, 289, 291 and 300) were substituted with alanine (Ala) residues, all of the lysine residues did not participate in the TsENO-PLG surface interaction. These four lysine residues (90, 289, 291 and 300) of TsENO were identified as active residues for PLG binding (Table 1, Figures 4B and C). The molecular model of the quadruple mutant (Lys90Ala + Lys289Ala + Lys291Ala + Lys300Ala) TsENO was also constructed by I-TASSER, and 3D structural alignments between TsENO and its quadruple mutant were performed by SuperPose and Swiss-PdbViewer. Two molecules exhibited similar 3D structures, and the local RMSD between the backbones and the global RMSD were 2.63 Å and 3.30 Å, respectively (Figure 5).

**Table 1:** **Shortlisted human PLG-interacting lysine residues of TsENO**

Lysine residue	Hydrophobic effect	H-bond interaction	ASA (Å^2^)	Loss in ASA (Å^2^)	Δ^i^G (kcal/mol)
90	3	1	146.93	135.98	1.16
198	0	1	31.36	6.95	0.06
229	1	0	35.26	35.09	0.56
233	2	0	75.59	46.97	0.72
289	2	0	53.70	45.08	0.72
291	2	0	169.68	82.02	1.07
300	1	0	50.51	45.66	0.31

*ASA* accessible surface area (Å^2^), *ΔiG* solvation energy of the corresponding residue (kcal/mol).

### Expression and purification of rTsENO and M-rTsENO

**Figure 6:**
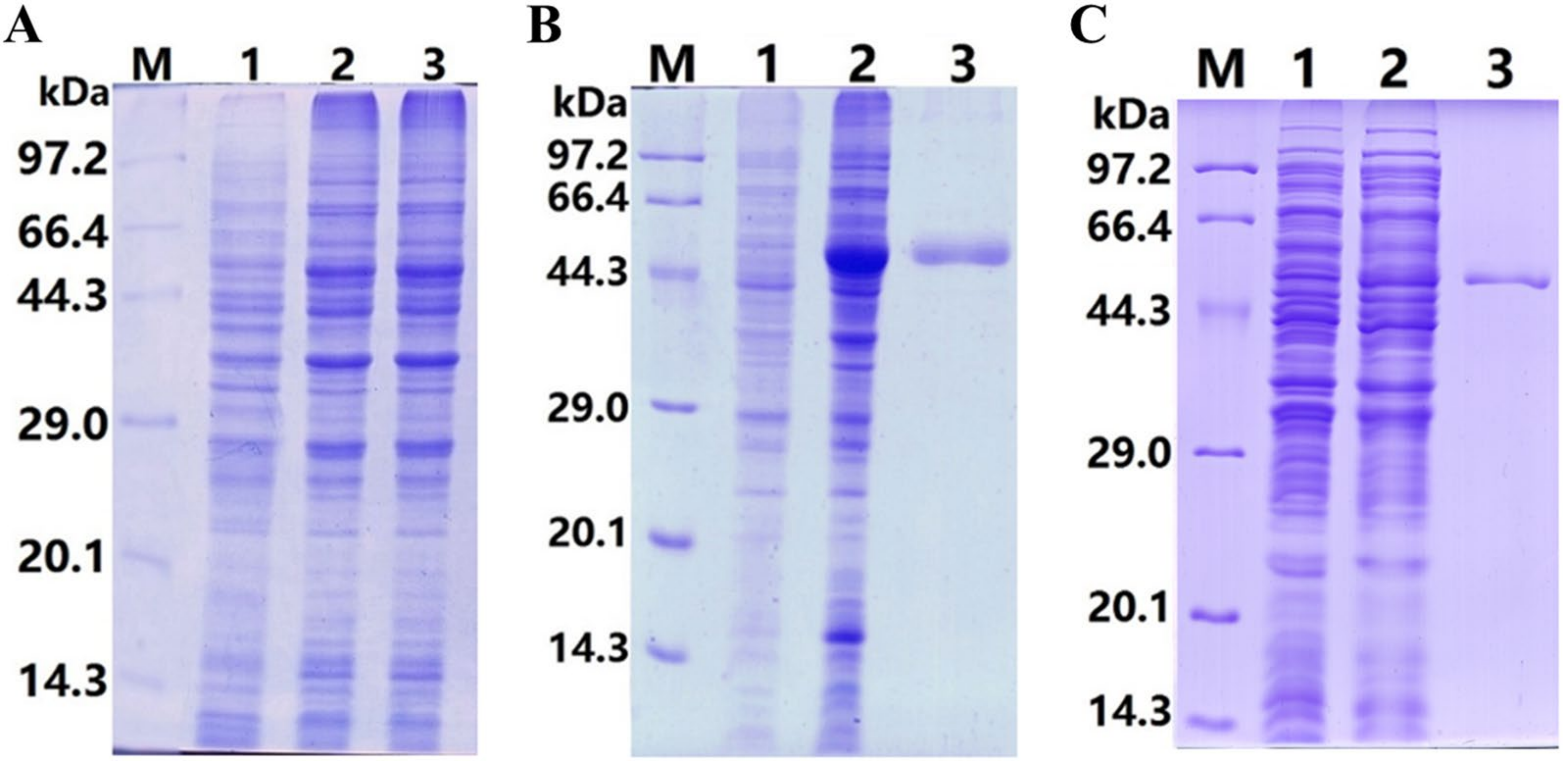
**Expression and purification of rTsENO and M-rTsENO. A** SDS-PAGE of rTsENO and M-TsENO expressed by recombinant plasmids. M: protein marker; 1: uninduced recombinant bacterial lysate; 2: induced recombinant pQE-80L/TsENO; 3: induced recombinant pQE-80L/M-TsENO. **B** SDS-PAGE of purified rTsENO. M: protein marker; 1: uninduced recombinant bacterial lysate; 2: induced recombinant pQE-80L/TsENO; 3: purified rTsENO (6 µg). **C** SDS-PAGE of purified M-rTsENO. M: protein marker; 1: uninduced recombinant bacterial lysate; 2: induced recombinant pQE-80L/M-TsENO; 3: purified M-rTsENO (2 µg).

The synthesized coding sequences of TsENO and M-TsENO were successfully cloned into the pQE-80L vector. *E. coli* BL21 harbouring pQE-80L/TsENO and pQE-80L/M-TsENO were induced for 5 h with 0.5 mM IPTG at 30 °C. The molecular weights of rTsENO and M-rTsENO were identified by SDS-PAGE as 51.95 kDa and 51.78 kDa, consistent with the previous predictions of ProtParam (Figure 6).

### Transcription levels of Ts-*eno* in different stages of *T. spiralis* development

**Figure 7:**
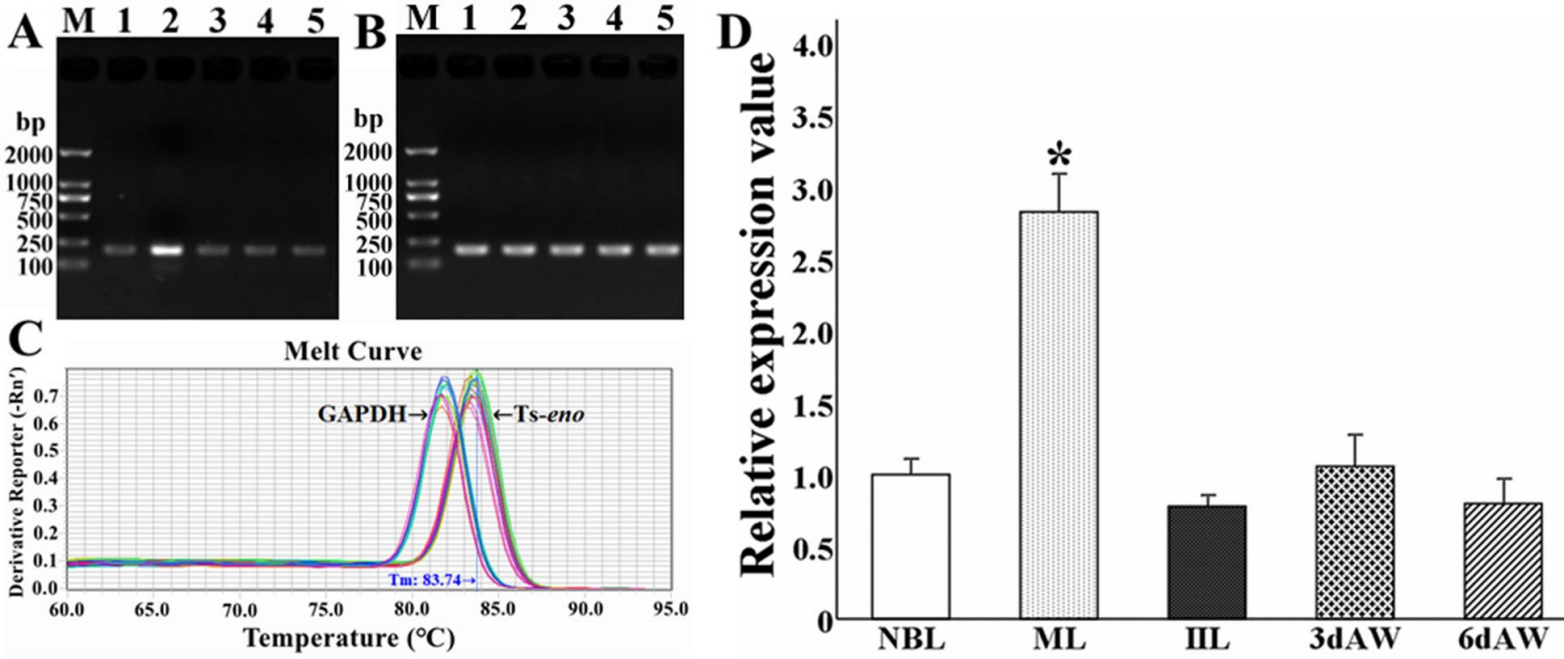
**Real-time quantitative PCR (qPCR) analysis of Ts-*eno*** **transcription at different stages of** ***T. spiralis*** **development. A** Agarose gel electrophoresis of the Ts-*eno* amplicon (188 bp). M: DL2000 DNA marker; 1: NBL; 2: ML; 3: IIL; 4: 3 days AW; 5: 6 days AW. **B** Agarose gel electrophoresis of the GAPDH amplicon (196 bp). M: DL2000 DNA marker; 1: NBL; 2: ML; 3: IIL; 4: 3 days AW; 5: 6 days AW. **C** Melt curves of Ts-*eno* and GAPDH generated by 7500 Software (version 2.0.5). **D** qPCR analysis of Ts-*eno* transcription at different *T. spiralis* stages. The asterisks (*) represent significant differences with other stages (*P* < 0.05).

qPCR analysis was carried out to quantify the relative transcriptional level of Ts-*eno* across different stages of *T. spiralis* development (ML, IIL, 3 days AW, 6 days AW and NBL). The qPCR results revealed that Ts-*eno* transcription was observed at all stages of *T. spiralis* development. The 2% agarose gel electrophoresis results revealed that the Ts-*eno* and GAPDH amplicons had sizes of 188 bp and 196 bp, respectively. The melt curves of Ts-*eno* and GAPDH generated by 7500 Software (version 2.0.5) showed only one peak for each amplicon, which indicated that the PCR products possessed very high specificity. The Ts-*eno* relative transcriptional level in ML was significantly higher than that in other developmental stages (IIL, 3 days AW, 6 days AW and NBL) (*F* = 7.878*, P* < 0.05) (Figure 7).

### Immunolocalization of TsENO

**Figure 8:**
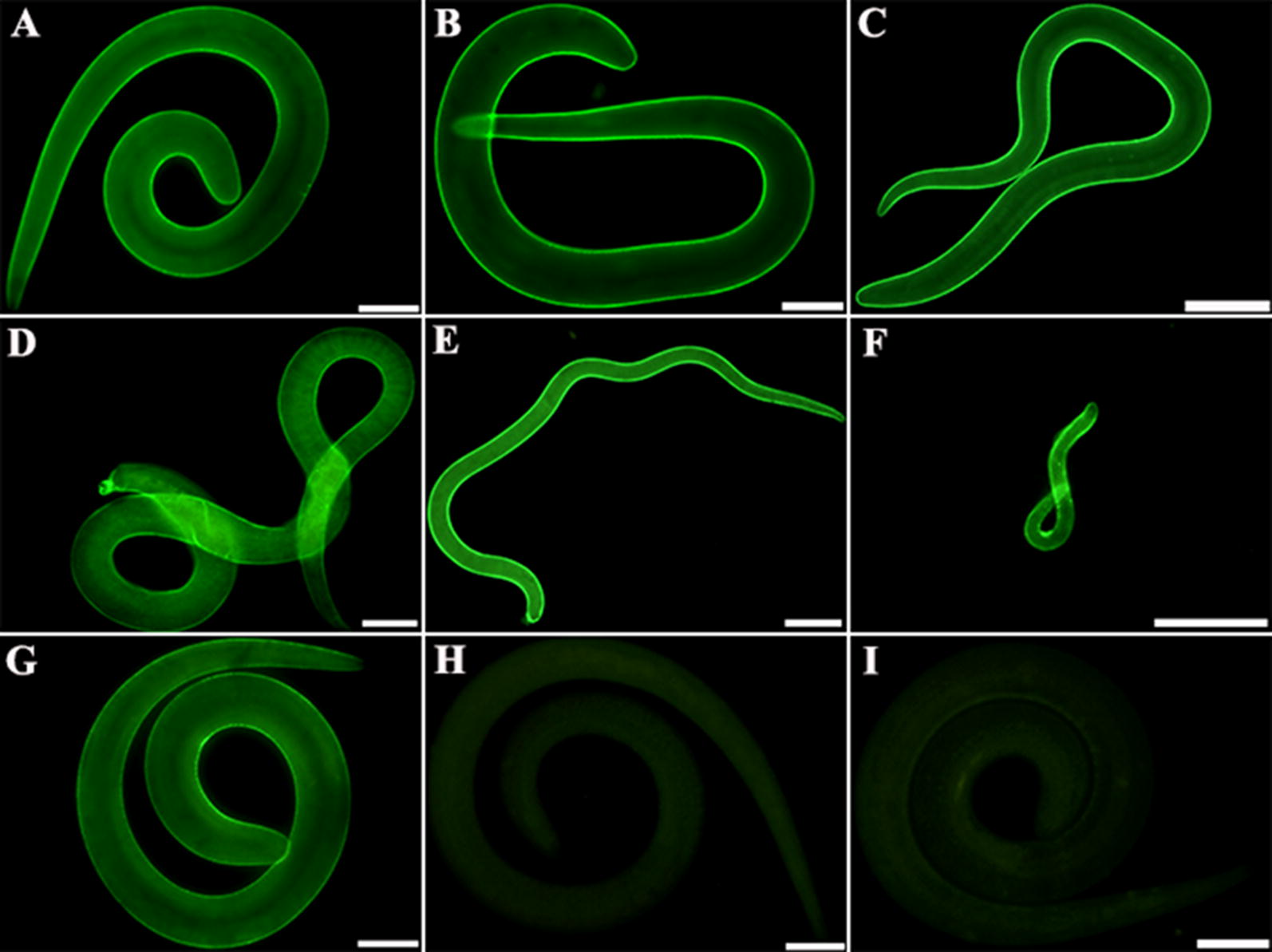
**Immunolocalization of TsENO at different stages of** ***T. spiralis*** **development. A**–**F** IFA with intact worms detected by anti-rTsENO serum. There was bright fluorescence staining on the cuticles of ML (**A**), 6 h IIL (**B**), 24 h IIL (**C**), 3 days AW (**D**), 6 days AW (**E**) and NBL (**F**). **G** ML detected by infectious serum served as the positive control. **H**–**I** ML detected by PBS and normal mouse serum were used as negative controls. Scale bars: 50 μm.

IFA of intact *T. spiralis* worms detected by anti-rTsENO serum showed that bright green immunofluorescence staining was observed on the epicuticles of *T. spiralis* in all life cycle stages. There was bright fluorescence staining on the cuticles of ML, 6 h IIL, 24 h IIL, 3 days AW, 6 days AW, and NBL, as probed by anti-rTsENO serum, whereas no staining was detected on the cuticles of ML incubated with normal mouse serum and PBS (Figure 8).

### Western blot validation of the PLG binding and interacting residues

**Figure 9:**
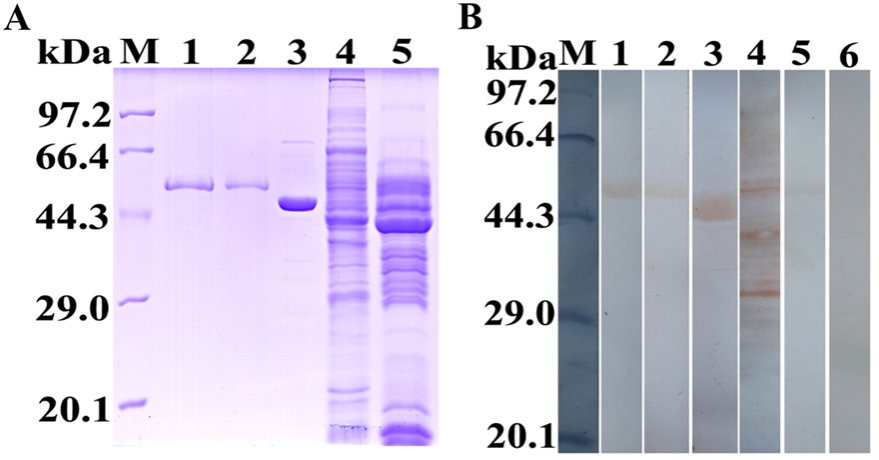
**Western blot analysis of rTsENO and M-rTsENO binding to human PLG. A** SDS-PAGE of rTsENO, M-rTsENO, ScENO, soluble antigens of ML and ES antigens of ML. M: protein marker; 1: rTsENO; 2: M-rTsENO; 3: ScENO; 4: soluble antigens of ML; 5: ES antigens of ML. **B** Western blot analysis of TsENO and M-rTsENO binding to human PLG. M: protein marker; 1: rTsENO; 2: M-rTsENO; 3: ScENO; 4: soluble antigens of ML; 5: ES antigens of ML; 6: BSA. The proteins mentioned above were blotted on a NC membrane and pre-incubated with human PLG. Subsequently, the membrane was incubated with an anti-PLG antibody. ScENO and BSA served as the positive control and negative control, respectively. The bands representing PLG binding to rTsENO (52 kDa), M-rTsENO (52 kDa), ScENO (47 kDa), soluble antigens of ML (26.9–82.9 kDa, including 52 kDa) and ES antigens of ML (52 kDa) were observed on the strips. The band representing PLG binding of rTsENO was more distinct than that of M-rTsENO, although the loaded amount of proteins was consistently 20 µg. There were some other unnamed PLG binding proteins with molecular masses ranging from 26.9 to 82.9 kDa among ML soluble antigens.

For Western blot analysis, the same amount of purified rTsENO and M-rTsENO, as well as ScENO, ML soluble antigens and ML ES antigens, were separated by SDS-PAGE and transferred to NC membranes. As confirmed by Western blot analysis, purified rTsENO, M-rTsENO and positive control ScENO were specifically recognized by the anti-PLG antibodies, whereas the negative control protein BSA was not. ML ES antigens also interacted with human PLG and showed the same binding band as rTsENO at the same position near 52 kDa. The ML soluble antigens contained many proteins that were capable of binding PLG under the same conditions. More than 10 major PLG-binding proteins, with approximate molecular masses ranging from 26.9 to 82.9 kDa, including the specific 52 kDa band of TsENO, were observed among the ML soluble antigens (Figure 9).

### ELISA validation of the PLG binding and interacting residues

**Figure 10:**
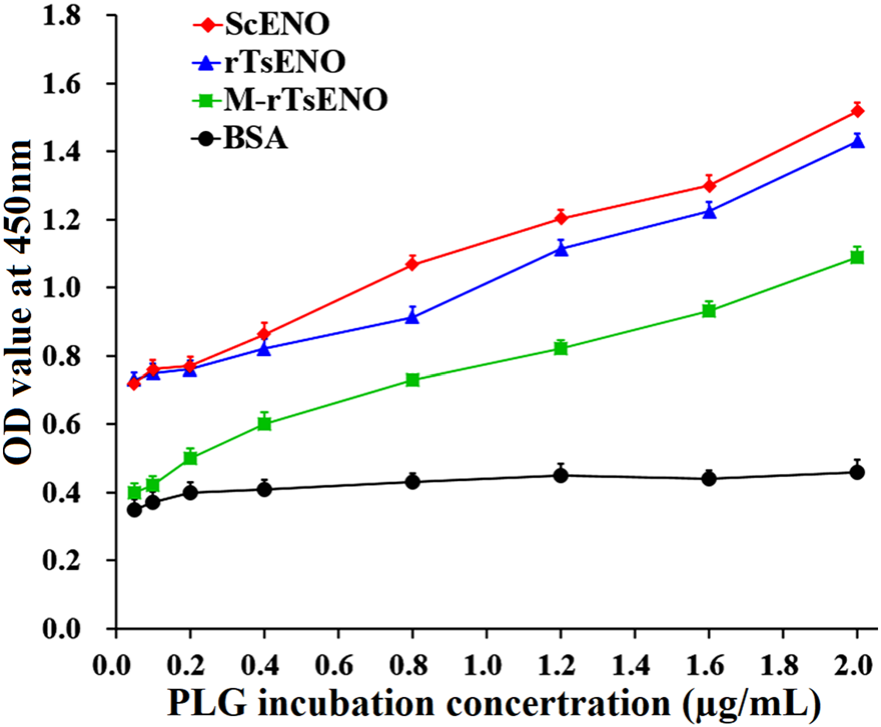
**PLG binding ability of rTsENO and M-rTsENO, as determined by ELISA.** ELISA plates were coated with rTsENO (0.6 μg/well), M-rTsENO (0.6 μg/well), ScENO (0.4 μg/well, positive control) or BSA (0.8 μg/well, negative control). Then, the wells were incubated with various concentrations of human PLG (0.05-2.0 µg/mL). An anti-PLG antibody was utilized to detect PLG under standard ELISA protocols. The ability of PLG to bind with rTsENO, M-rTsENO and ScENO was dose-dependent. The dots on the different lines indicate the mean absorbance values at 450 nm $({\bar{\text{x}}} \pm {\text{SD}}).$

The binding ELISA results confirmed that rTsENO was capable of binding human PLG and displayed an increasing trend with increasing PLG concentrations (*F* = 1850.78, *P* < 0.05). In addition, the OD values of M-rTsENO and ScENO appeared to exhibit a dose-dependent ascending pattern (*F*_M-rTsENO_ = 1725.65, *F*_ScENO_ = 2397.07; *P* < 0.05). According to the *t* test, the OD values of the rTsENO groups were always higher than those of the M-rTsENO groups at different PLG concentrations (*t*_1_ = 40.42, *t*_2_ = 40.41, *t*_3_ = 31.84, *t*_4_ = 26.94, *t*_5_ = 26.57, *t*_6_ = 35.52, *t*_7_ = 25.13, *t*_8_ = 41.63; *P* < 0.05). Compared with rTsENO, M-rTsENO showed a marked loss of PLG binding ability, with a decrease ranging from 20.04% to 45.37%. Notably, the quadruple mutant (Lys90Ala + Lys289Ala + Lys291Ala + Lys300Ala) M-rTsENO showed a decrease in binding with human PLG of nearly 45.37% when the concentration of PLG was 0.05 µg/mL. Non-significant differences between rTsENO and ScENO were detected at lower PLG concentrations (0.05, 0.1 and 0.2 µg/mL). However, the binding ability of ScENO was slightly stronger than that of rTsENO at higher PLG concentrations (0.4, 0.8, 1.2, 1.6, 2.0 μg/mL). BSA showed no interaction with human PLG under the same experimental conditions (Figure 10).

## Discussion

Enolase, also known as phosphopyruvate hydratase, was discovered in 1934 by Lohman and Mayerhof [46]. It is a pivotal enzyme in glycolysis that catalyses the dehydration of 2-phosphoglycerate (2-PGA) to phosphoenolpyruvate (PEP). While enolase is a ubiquitous metalloenzyme involved in glycolysis, it also seems to be an abundantly expressed cytosolic protein in many organisms [20, 47]. Thus, the transcription of Ts-*eno* was observed throughout the whole life cycle of *T. spiralis*. Previous studies have shown that both the tricarboxylic acid cycle (Krebs cycle) and glycolysis exist in *T. spiralis* AW and ML [48, 49]. However, the most important metabolic pathway in ML seems to be an improved approach to classical glycolysis [50, 51]. Much more TsENO is used by ML because ML reside in the capsule in an anaerobic environment. In fact, the relative Ts-*eno* transcription level was significantly higher in ML than in the other phases.

Unlike the expression and subcellular localization of other housekeeping glycolytic enzymes, the expression and subcellular localization of enolase have been found to vary under various pathophysiological conditions [20]. In addition to its classical glycolytic enzyme function, enolase appears to be located on the surface of different kinds of pathogens, where it serves as a PLG receptor, concentrating PLM activity and facilitating invasion [14, 18, 20, 21]. It has been shown that a strong relationship exists between enolase and the host’s fibrinolytic system during invasion [18]. The binding between the host’s PLG and helminth enolases has been studied in recent years, and the results have indicated that the PLG-enolase interaction requires lysine residues [52]. Full-length PLG comprises seven domains: N-terminal Pan-apple domain (PA), kringle domains 1–5 (KR 1–5) and serine protease domain (SP) [8]. Interactions of the host’s PLG or its isolated kringle domains with lysine residues occur in KR 1, 2, 4 and 5 [7]. Specific structural domains named lysine binding sites (LBS) are present in these kringle domains and provide binding sites for lysine residues of PLG receptors [6]. KR 1 and KR 4 possess the highest binding affinity for lysine-type ligands [53–56], while KR 2 exhibits the lowest affinity [57].

One possible mechanism for the enhanced PLG activation rates is that the LBS within KRs interact with lysine residues of proteins on pathogen surfaces to induce PLG to adopt a conformation that can be activated [7]. Hence, the lysine residues of receptors are essential for the initial recruitment and subsequent conformational change of PLG [8, 9]. Furthermore, recombinant enolase showed stable PLG binding and activating activity in some previous studies, indicating that enolase could play an important role in parasite invasion [58, 59]. For instance, enolases have also been reported as PLG receptors in the helminths *Onchocerca volvulus*, *Dirofilaria immitis*, *Schistosoma mansoni*, *Schistosoma bovis*, *Fasciola hepatica*, *Echinostoma caproni*, *Taenia solium*, *Taenia multiceps* and *Taenia pisiformis* [16, 58–65]. Among these enolases mentioned above, the enolases of *Schistosoma mansoni* and *Taenia pisiformis* were also demonstrated to promote PLG activation [16, 62]. Hence, TsENO may also play such a role in *T. spiralis* invasion by interacting with the host’s PLG.

Molecular docking analysis has not been carried out for the TsENO-PLG interaction in *T. spiralis*. To determine the TsENO-PLG binding sites in detail, the I-TASSER program was adopted to construct the 3D structures of TsENO and its mutant in the present study. Moreover, a series of bioinformatics software programs were applied to analyse the key residues involved in the interface between TsENO and PLG. Interestingly, lysine residues were the most frequently present amino acids on the binding surface. Although Lys198, Lys229 and Lys233 were identified in the interface, they were not the key residues for PLG binding, because the ASA loss for Lys198 was only 6.95 Å^2^, which was less than 10 Å^2^ [66]. In contrast, the PLG residues interacting with Lys229 and Lys233 were located in KR 3. Considering the lack of LBS in KR 3, Lys229 and Lys233 should not be the key residues for PLG binding [6, 7, 53–57]. Notably, one of the key lysine residues (90) binds to Ser383 (located in KR 4 of PLG) by forming hydrogen bonds. The other three key lysine residues (289, 291 and 300) were also involved in hydrophobic contacts with KR 4 and the SP of PLG. These four lysine residues (90, 289, 291 and 300) of TsENO were considered to be active residues for PLG interaction. Then, we confirmed the bioinformatics prediction using site‐directed mutagenesis by substituting lysine residues with neutrally charged alanines. Although the 3D structural alignments identified only slight differences between TsENO and its quadruple mutant, the decrease in PLG binding activity was significant. Hence, the reduced binding ability can be attributed to the substitution of four key lysine residues (Lys90Ala, Lys289Ala, Lys291Ala and Lys300Ala). As observed in the ELISA, the TsENO mutant lost almost half (45.37%) of its binding ability, suggesting that these lysine residues might be crucial in the TsENO-PLG interaction.

In summary, we identified TsENO as a PLG receptor based on the results of computational methods and experimental techniques. The qPCR results showed that the Ts-*eno* transcription level in ML was significantly higher than in the other stages. The IFA results revealed that TsENO is expressed on the cuticle surface of *T. spiralis* throughout its life cycle. Furthermore, TsENO was identified by Western blotting among ES antigens of ML, consistent with the signal peptide prediction. These findings—the Ts-*eno* transcription level in ML and the presence of TsENO among ES antigens of ML—were somewhat different from those of one previous study on TsENO [67]. The varying lengths of TsENO sequences adopted in different studies may account for this diversity. In view of the unique mode of metabolism in the ML stage [48–51] and the protective immunity induced by enolase [68], the results of this study are still reliable and helpful for understanding the relationship between *T. spiralis* and its host.

Our results indicated that TsENO was capable of contact with the host’s PLG either expressed on the body surface of the worm or presented in the ES products. Previous studies have shown that helminth enolases enhanced the activation of the host’s PLG and that PLG-mediated proteolysis contributed to larval invasion and migration [14–21, 58–65]; hence, TsENO could accelerate PLG activation and *T. spiralis* invasion of the host’s intestinal wall. Unfortunately, the molecular mechanism of PLG activation by enolase and subsequent degradation of ECM is still unclear. Further studies based on in vitro and in vivo experiments should be carried out to elucidate this interesting function of TsENO in the process of larval invasion.

In conclusion, our results revealed that TsENO has strong binding activity with the host’s PLG. Four lysine residues (90, 289, 291 and 300) of TsENO play an important role in PLG binding and could accelerate PLG activation and *T. spiralis* invasion of the host.

## Abbreviations

AW: adult worms
BSA: bovine serum albumin
3D: three-dimensional
ECM: extracellular matrix
ELISA: enzyme-linked immunosorbent assay
ES: excretory-secretory
FITC: fluorescein isothiocyanate
HRP: horseradish peroxidase
IFA: immunofluorescence assay
IIL: intestinal infective larvae
IPTG: isopropyl β-d-1-thiogalactopyranoside
I-TASSER: Iterative Threading ASSEmbly Refinement
KR: kringle domain
LBS: lysine binding sites
ML: muscle larvae
M-rTsENO: mutant recombinant *T. spiralis* enolase
M-TsENO: mutant *T. spiralis* enolase
NBL: newborn larvae
NC: nitrocellulose
OD: optical density
PBS: phosphate-buffered saline
PBST: phosphate-buffered saline containing tween
PDB: protein data bank
PLG: plasminogen
PLM: plasmin
RMSD: root mean square deviation
rTsENO: recombinant *T. spiralis* enolase
ScENO: *S. cerevisiae* enolase
SD: standard deviation
SDS-PAGE: sodium dodecyl sulfate–polyacrylamide gel electrophoresis
SP: serine protease domain
TBST: tris-buffered saline containing Tween
Ts-*eno*: *T. spiralis* enolase gene
TsENO: *T. spiralis* enolase
